# Options for acquiring motherhood in absolute uterine factor infertility; adoption, surrogacy and uterine transplantation

**DOI:** 10.1111/tog.12729

**Published:** 2021-03-19

**Authors:** Benjamin P Jones, Niccole Ranaei‐Zamani, Saaliha Vali, Nicola Williams, Srdjan Saso, Meen‐Yau Thum, Maya Al‐Memar, Nuala Dixon, Gillian Rose, Giuliano Testa, Liza Johannesson, Joseph Yazbek, Stephen Wilkinson, J Richard Smith

**Affiliations:** ^1^ Clinical Research Fellow Department of Surgery and Cancer Imperial College London Du Cane Road London W12 0NN UK; ^2^ Specialty Trainee in Obstetrics and Gynaecology Queen Charlotte’s & Chelsea Hospital Imperial College NHS Trust London W12 OHS UK; ^3^ Research Associate in Ethics Department of Politics, Philosophy and Religion Lancaster University Lancaster LA14YQ UK; ^4^ Gynaecology Oncolology Subspecialty Trainee Hammersmith Hospital Imperial College NHS Trust London W12 OHS UK; ^5^ Fertility Specialist The Lister Fertility Clinic London SW1W 8RH UK; ^6^ Specialty Trainee in Obstetrics and Gynaecology Queen Charlotte’s & Chelsea Hospital Imperial College NHS Trust London W12 OHS UK; ^7^ Clinical Nurse Specialist Queen Charlotte’s & Chelsea Hospital Imperial College NHS Trust London W12 OHS UK; ^8^ Consultant Gynaecologist Queen Charlotte’s & Chelsea Hospital Imperial College NHS Trust London W12 OHS UK; ^9^ Transplant Surgeon Baylor University Medical Center Dallas Texas 75246‐2088 USA; ^10^ Gynaecology Oncology Surgeon and Medical Director of Uterus Transplant Baylor University Medical Center Dallas Texas 75246‐2088 USA; ^11^ Consultant Gynaecologist Hammersmith Hospital Imperial College NHS Trust London W12 OHS UK; ^12^ Professor of Bioethics Department of Politics, Philosophy and Religion Lancaster University Lancaster LA14YQ UK

**Keywords:** adoption, infertility, surrogacy, transplantation, uterus

## Abstract

**Key content:**

- Following the diagnosis of absolute uterine factor infertility (AUFI), women may experience considerable psychological harm as a result of a loss of reproductive function and the realisation of permanent and irreversible infertility.
- Adoption enables women with AUFI, and their partners, to experience social and legal parenthood, also often providing benefits for the adopted child.
- Surrogacy offers the opportunity to have genetically related offspring. Outcomes are generally positive in both surrogates and the children born as a result.
- Uterine transplantation is the only option to restore reproductive anatomy and functionality. While associated with considerable risk, it allows the experience of gestation and the achievement of biological, social and legal parenthood.

**Learning objectives:**

- To gain an understanding of the routes to parenthood available for women with AUFI experiencing involuntary childlessness, such as adoption, surrogacy and, most recently, uterine transplantation
- To consider a suggested management plan to facilitate counselling in women with AUFI who experience involuntary childlessness.

**Ethical issues:**

- In the UK, while the number of children requiring adoption continues to increase, the number being adopted from care is decreasing.
- Some cultures may hold ethical or religious beliefs that surrogacy is unacceptable, and its legal position in many jurisdictions is problematic.
- Restrictive selection criteria and high costs may limit future availability of uterine transplantation

## Introduction

Absolute uterine factor infertility (AUFI) is a form of infertility whereby conception and/or maintenance of pregnancy is impossible owing to uterine absence or dysfunction. AUFI may be congenital, such as in Mayer–Rokitansky–Küster–Hauser (MRKH) syndrome; acquired, following hysterectomy; or from the development of uterine pathology, such as severe Asherman’s syndrome. Regardless of aetiology, the diagnosis of AUFI is often sudden and unexpected, coming after investigation for primary amenorrhea, hypomenorrhea, or following urgent or unplanned hysterectomy. Others, such as those with severe Asherman’s syndrome, may be diagnosed after years of poor reproductive history, often following numerous unsuccessful hysteroscopic procedures. After diagnosis, women with AUFI experience the loss of reproductive function and the realisation of permanent and irreversible infertility, which is associated with considerable long‐term emotional burden.^1^, ^2^ Management of AUFI thus requires an integrated, multidisciplinary approach, involving gynaecologists, psychologists and clinical nurse specialists.^3^ Additionally, particularly in conditions such as MRKH, when the diagnosis commonly occurs during adolescence, counselling and patient support groups can be particularly beneficial.^4^

After a diagnosis of infertility, many women experience anxiety, depression, low self‐esteem, loss of gender identity, a decrease in their quality of life and an enduring sense of incompleteness and grief.^5^, ^6^, ^7^, ^8^ Worse psychological outcomes arise in women experiencing infertility who fail to conceive after assisted reproductive technology (ART) treatment than in those who are successful.^9^ In low income and/or strongly pronatalist cultures and societies, there may also be associated socioeconomic implications arising from an infertility diagnosis, including a negative effect on social status and worsening marital discourse.^10^

While childlessness, or remaining ‘child‐free’, is a choice increasingly made by both genders,^11^ most women still expect to acquire motherhood by conceiving without medical assistance, carrying a pregnancy themselves and giving birth to their own children. However, women with AUFI who seek parenthood have – until recently – had no option but to change their reproductive plans and either accept involuntary childlessness or acquire parenthood through adoption or surrogacy. After more than 70 uterine transplantation (UTx) procedures worldwide and at least 18 live births,^12^ women with AUFI may soon be able to access an alternative route to parenthood that would allow them to conceive, gestate and give birth to their own children. However, despite the additional benefits it promises, UTx is associated with considerable risk and currently necessitates conception via in vitro fertilisation (IVF), a highly medicalised pregnancy and delivery by caesarean section.

This review explores the options available for women with AUFI to acquire motherhood, discusses the advantages and disadvantages of each option and provides a suggested management algorithm for women with AUFI who experience involuntary childlessness, based on individual reproductive aspirations.

## Adoption

Adoption is the permanent transfer of parental rights and responsibility from a child’s birth parents to adoptive parents, creating a new family unit that will raise the child. For women with AUFI who seek parenthood, adoption benefits include social and legal parenthood and an opportunity to enhance the lives of children whose genetic parents are unable to care for them.^13^ In the UK, the number of children defined as being under the care of local authorities has increased every year since 2013. This is primarily associated with an increased number of care orders, resulting in 78 150 children in care in 2018/19. In contrast to this rise, the number of children who are adopted from care continues to decrease, with just 3570 adoptions in the same period.^14^

While adoption is usually a mutually beneficial arrangement for both parents and their adopted children, it is often associated with several challenges or attachment‐related difficulties that require consideration for prospective parents. Of all children who are looked after by local authorities, 63% have previously experienced abuse or neglect.^14^ Adopted children are more likely to be diagnosed with emotional, behavioural and relational difficulties and^15^, ^16^ to access mental health services in the future,^13^ and fare worse in terms of academic attainment^17^ compared with children under the guardianship of their birth parents. Adverse outcomes extend into adulthood.^18^ However, successful placements with adoptive families have resulted in better psychological development and wellbeing outcomes for previously looked‐after children, especially when adopted at a younger age.^19^, ^20^, ^21^

Potential adopters may find adopting a daunting prospect. It can be a very lengthy process, typically including a formal evaluation process involving references, background checks and home visits, before a training period and a more detailed assessment, while the adoption agency seeks a good match between child and potential adopters. In the UK, this matching process can take up to 2 years^22^ and is by no means guaranteed. There is the additional insecurity that the child may not even subsequently be relinquished from their birth parents. Initial reports portrayed outcomes for adoptive parents to be inferior to biological ones, with suggestions of increased anxiety, anger, grief and inability to bond.^23^, ^24^ However, more recent studies have suggested positive outcomes for parents following adoption, with three‐quarters of adoptive parents reporting a positive effect on their family.^25^, ^26^

The realities of adoption are undoubtedly associated with numerous challenges. This is exemplified by a recent unpublished survey from almost 2700 adopters, undertaken in collaboration with Adoption UK.^27^ More than one‐quarter of parents responding to this survey described serious effects on the wider family, or that their wider family relationships were at risk or had already been disrupted. Around half of respondents found it challenging but stable and one‐quarter purported it to be fulfilling and stable. Despite almost two‐thirds reporting aggressive behaviour towards them from their child, most (88%) were glad that they adopted. Another study identified that 9–13% of adoptions broke down and 21–25% were finding it difficult,^28^ further highlighting the challenges faced by adoptive families. Unrealistic expectations, particularly with regards to subsequent academic achievement, have also been identified as factors affecting adjustment.^29^ From a psychological perspective, adoptive parents have reported similarly positive depression, self‐esteem and wellbeing scores when compared with biological parents.^30^

Cross‐border adoption entails the legal adoption of children born in other countries. These account for approximately 30 000 adoptions worldwide per year. Cross‐border adoption offers the opportunity for vulnerable children, mostly from low‐income, undeveloped countries, to be raised in a wealthier country, with better healthcare, education and opportunities. However, whereas there is unquestionable opportunity for great benefit, considerable challenges remain in relation to safeguarding and exploitation, including the potential for the illicit movement of vulnerable children who have been illegally separated from their families. Further issues stimulating debate relate to the cultural identity of children following cross‐border adoption.^31^

## Surrogacy

Surrogacy is the process whereby a woman (the surrogate) gestates and gives birth with a pre‐arranged plan of giving the child to another person or couple: the ‘intended’ parents. Surrogacy arrangements can be paid (‘commercial’) or unpaid (‘altruistic’). They are also commonly divided into ‘full’, or ‘straight’ or ‘traditional’, surrogacy arrangements, and ‘host’, or ‘gestational’, surrogacy. In full surrogacy, the surrogate provides her own eggs, so is genetically related to the child. In host surrogacy, she does not; the eggs may come either from the intended parents or an egg donor. The occurrence of AUFI provides a strong prima facie justification for utilising surrogacy.^32^ In such women, gestational surrogacy is considerably more prevalent than full surrogacy because, subject to satisfactory ovarian reserve, it allows them to be biologically related to their children. Thousands of children have now been born using surrogacy arrangements.^33^ However, some cultures or families may still hold ethical or religious beliefs that surrogacy is unacceptable. Furthermore, surrogacy’s legal position in many jurisdictions is problematic.

**Figure 1:**
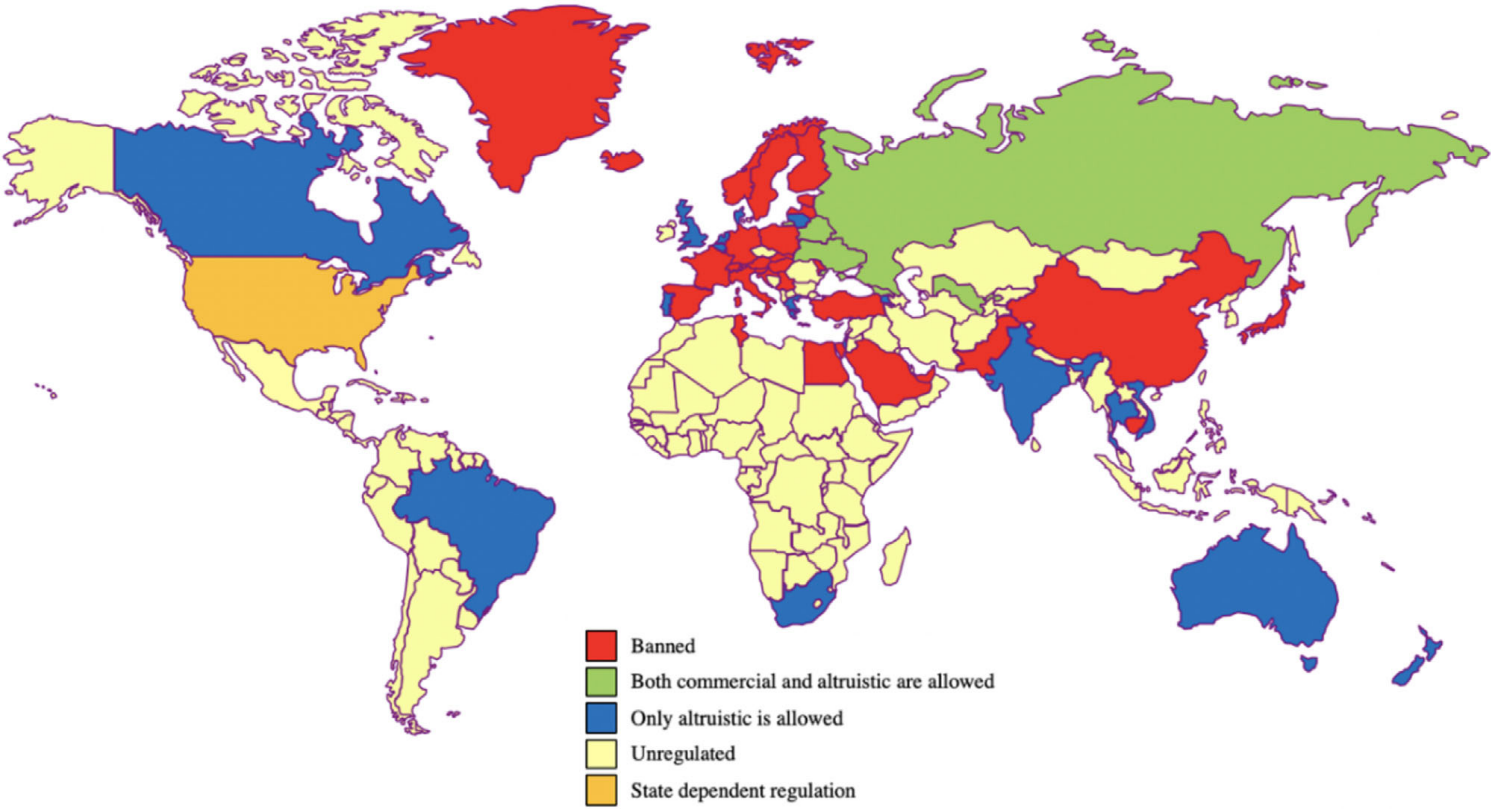
International variation of surrogacy law.

Surrogacy regulation varies internationally and between US states, as represented in Figure 1. Paid, commercial surrogacy is permitted and legally enforceable in certain countries including Russia, Ukraine and Georgia. In other countries, only unpaid, altruistic surrogacy is permitted, with paid arrangements and their brokerage being forbidden. Countries where this applies include the UK, Australia, Canada, Brazil, India and South Africa. In many areas of the world, including most of Western Europe, China, Japan, Pakistan, Turkey, Saudi Arabia and some areas of North America, restrictive legislation explicitly or effectively forbids all forms of surrogacy. Thus, it is excluded as a possibility for more than one‐third of the world’s population. A recent survey orchestrated by the International Federation of Fertility Societies (IFFS), which included respondents from 65 countries, reported that surrogacy was permitted by statute or guideline in just 38% of the countries represented, and prohibited in 56%.^34^

Although the UK was one of the first countries to introduce a regulatory framework for ART, subsequent legislative reforms have received criticism.^35^ The Surrogacy Arrangements Act 1985 was heavily influenced by recommendations from the Committee of Inquiry into Human Fertilisation and Embryology 1984, referred to as the Warnock Report.^36^ The Warnock Report highlighted concerns about the potential use of financial incentives in surrogacy commercialisation to exploit vulnerable women. Central to the Surrogacy Arrangements Act 1985 was the prohibition of commercial surrogacy. However, no safeguards were put in place to protect intended parents or surrogates and the welfare of subsequent children was not addressed. Such safeguards were not put in place until the enactment of the Human Fertilisation and Embryology Act 1990, which provided a legal framework for transfer of parental rights from surrogates to the intended parents and incorporated a welfare principle.

Surrogacy is permitted in the UK, but surrogacy agreements are not legally enforceable. This means that the surrogate will be the child’s legal mother at birth, regardless of the origin of the gametes that created the embryo. If the surrogate is married, then her husband, who is biologically unrelated to the child, would automatically be considered the legal father. The surrogate can then transfer legal parenthood to the intended parents 6 weeks after birth of the child. Although cases in which surrogates decide not to relinquish the child are rare, this legal position carries some risk for the intended parents. The possibility of the surrogate not cooperating with the transfer of parental rights after birth may generate anxiety and make surrogacy less appealing as a reproductive option.^37^ For the surrogate, there is also a risk that intended parents may renege on the agreement, leaving her to take care of the child, especially in the event that the child is born with a disability or medical conditions. In disputes between intended parents and the surrogate, the courts will decide based on the child’s best interests; the child’s rights are deemed to be paramount in such cases, in line with the Children Act 1989 (England and Wales). However, at the time of writing, there is increasing pressure within the UK to review legislation so that genetic parents assume legal rights at birth.^38^

While domestic surrogacy rates in the UK have remained relatively stable in recent years, a growing minority of prospective parents are utilising cross‐border surrogacy.^35^, ^37^ This increase has been attributed to less restrictive, or clearer, regulations abroad, in addition to the difficulty of finding a surrogate domestically, especially when payment is limited or prohibited.^39^, ^40^ However, utilising international surrogates does not bypass UK surrogacy legislation. Not only may issues surrounding the child’s legal recognition complicate attempts by the intended parents to travel home, but they are still required to apply for a parental order upon their return to the UK to become the child’s legal parents.^41^ Critics have also suggested that, from an ethical standpoint, cross‐border commercial surrogacy from low‐income countries is particularly problematic. Concerns centre around the surrogates’ autonomy and wellbeing, in addition to the potential for such arrangements to be exploitative. Major worries expressed here are that surrogates from low‐income countries may be ‘coerced by poverty’, which invalidates their consent, and they are likely to be underpaid and maltreated by intended parents or commercial intermediaries.^41^, ^42^ However, some cross‐border surrogates have reported positive experiences. It could even be argued that surrogacy is a less exploitative and less harmful means of earning money than other available opportunities.^43^

UK surrogates may be compensated with reasonable expenses only. A 2018 report by Surrogacy UK stated that the mean average compensation for domestic surrogacy at that time was £10,694.13; the highest reported in this survey was £23,500.^44^ Higher amounts were made for some international surrogacy arrangements between the USA and the UK, with one involving a payment of £96,000.^44^ So far, courts have usually taken a permissive view of relatively high expenses payments, with legal parenthood often being granted provided that it is perceived to be in the child’s best interests. A recent cross‐sectional study suggests that the average cost of surrogacy in the UK is approximately £25,000. However, the costs associated with surrogacy vary dramatically internationally; in the USA, the median associated cost was found to be £120,000.^39^

When considering the long‐term outcomes in children born to surrogates, a recent systematic review revealed similar perinatal outcomes to IVF with oocyte donation.^37^ Moreover, there are no major differences in psychological development compared with children born to nonsurrogates.^37^ A 10‐year prospective study in the UK showed that families usually maintain good relationships with surrogate families. Most children were aware how they were conceived and did not suffer negatively as a consequence.^45^

The outcomes in surrogate mothers are also largely encouraging, with most reporting positive experiences. Analysis of 16 studies assessing long‐term psychological outcomes found no long‐lasting, serious psychopathology.^37^ However, some surrogates found it difficult to relinquish care of their born child to the intended parents.^46^ One study, in particular, demonstrated that more than one‐third (35%) of surrogate mothers had such difficulties, although this reduced to 6% after 12 months.^46^ Similarly, when considering long‐term psychological outcomes of intended mothers and their relationships with their children, no major differences were shown when compared with mothers who conceive naturally.^37^

## Uterine transplantation

UTx entails transplantation of the uterus, including the cervix, as well as the surrounding ligamentous tissues and supplying and draining blood vessels. UTx is the only therapeutic intervention that restores reproductive anatomy and functionality in women with AUFI. It not only enables the experience of gestation, but allows biological, social and legal parenthood, thereby avoiding some of the potential problems with surrogacy discussed above.

In 2014, the first live birth following UTx was achieved in Sweden.^47^ This was achieved after a series of nine UTx procedures, which demonstrated the procedure’s feasibility using living donors.^48^ Eight live births have since been reported from this pivotal study,^49^ the success of which has paved the way for UTx procedures to be undertaken globally. The first live birth following UTx using a deceased donor was subsequently achieved in Brazil in 2017.^50^ While the details from several cases remain unpublished, a recent review of 45 UTx cases reported at least 18 live births^12^ and at least double this figure has been reported in the media, demonstrating that UTx is unquestionably feasible. However, more than one‐quarter of cases required emergency hysterectomy and an additional 10% suffered complications necessitating further surgical intervention, thus highlighting the considerable associated risk involved.^12^

UTx can be undertaken using either living or deceased donors. Each donor type presents differing advantages and disadvantages,^51^ and has distinct ethical implications.^52^, ^53^ Using living donors has organisational advantages, including plentiful time to assess the recipient and donor preoperatively, as well as arrange the highly skilled multidisciplinary team required to undertake the operation. While it is currently not possible to evaluate clinical and reproductive outcomes in UTx cases between donor type, evidence shows that clinical outcomes in other solid organ transplants are better when living donors are used.^54^ However, the major advantage of using deceased donors is that risk to the donor is completely removed. In cases of living donor UTx so far, more than 1 in 10 donors have suffered a complication necessitating further surgical intervention,^12^ which highlights the risk involved when using living donors.

Immunosuppression after UTx is essential and intensive follow‐up is required to assess recovery, while monitoring for rejection and immunosuppression‐related complications. Histological assessment of cervical biopsies is currently the only reliable method to detect rejection.^48^, ^55^, ^56^ After 6–12 months, following stabilisation on a nonteratogenic immunosuppression regimen, embryo transfers can be commenced.^57^ Using a single euploid blastocyst is recommended to optimise the probability of IVF success, while reducing the risk of multiple gestation.^12^ Following conception, high‐risk pregnancy care should ensue, with expert maternofetal medicine input, with a view to deliver by caesarean section at 37 weeks of gestation, unless clinically indicated sooner. While consideration should be given to the risks of late preterm/early term delivery, such as transient tachypnoea of the newborn (TTN) and potentially inferior cognitive outcomes,^58^, ^59^ the potential for painless labour brings potentially greater – albeit difficult to quantity – risk, with concerns regarding the structural integrity of the graft and how the vascular anastomoses would fare, following onset of contractions. Following birth, depending on reproductive plans and clinical condition, further embryo transfers can take place, or completion hysterectomy should be carried out. Following graft removal, transplant‐related medications and immunosuppression can be stopped, thereby reducing long‐term immunosuppression morbidity, such as infection and neoplasia.^60^, ^61^

UTx integrates complex bioethical debates from the fields of organ transplantation and assisted reproduction.^62^, ^63^ Topics examined have included the welfare of children born through UTx,^64^, ^65^ the values of reproductive autonomy and gestational parenthood,^66^, ^67^ comparisons between surrogacy and UTx^68^, ^69^ and broader questions surrounding publication, institutional requirements and research ethics.^70^ UTx has also attracted criticism because alternative pathways to motherhood exist.^71^ Some argue that if alternatives, such as adoption and surrogacy were presented and viewed more positively, then fewer women would seek UTx. It is also claimed that by providing UTx, undesirable attitudes towards parenthood might be reinforced and discriminatory social biases perpetuated; specifically, pronatalism (bias in favour of reproduction), gestationalism (bias in favour of gestational parenthood) and geneticism (bias in favour of genetic parenthood).^72^ These criticisms have also been specifically deployed against publicly funding UTx in countries with socialised medical care^73^, ^74^ and in insurance‐based or mixed systems.^75^ In this context, it has been argued that UTx improves on other options, such as surrogacy, only by satisfying personal desire to experience gestation and childbirth and that these are insufficient to justify the high financial cost associated with UTx, which has been estimated at almost €100,000 in European economies.^76^

These arguments, however, can be challenged. Firstly, it is not possible to generalise about how suitable adoption and surrogacy really are for women with AUFI. Their appropriateness depends on individual circumstance, taking account of personal values, religious and/or cultural background and the legal context. In most countries, even if not prohibited, surrogacy remains socially and legally complex. In such circumstances, despite the considerable associated risk, UTx may be a reasonable preference.^77^ Secondly, concerns about discriminatory social bias look more like a critique of reproductive medicine in general than a specific reason to not offer UTx. That said, UTx is presently more difficult to justify than IVF owing to the comparatively high costs and risk level.^62^, ^63^ Finally, it is difficult to ascertain why the mere existence of alternatives dictates the necessity to stop providing UTx. Interventions such as pinnaplasty, breast reconstruction after mastectomy and scalp cooling for chemotherapy are performed to enhance quality of life and protect people from hostile treatment for not conforming to prevailing norms. Arguments for UTx can be made on similar grounds and, even with alternatives available, UTx can be justified if it is in the woman’s interests.^78^

Perceptions of UTx among women with AUFI already appear very positive, despite the relative infancy of the procedure. A UK study demonstrated that 97.5% of women with AUFI would choose UTx over surrogacy and adoption, despite being aware of the additional risks posed by UTx.^3^ Another study, specifically assessing perceptions in women with MRKH, showed that almost two‐thirds of participants were motivated to undergo UTx, even after becoming aware of the associated risks.^79^ This is similar to the findings of a questionnaire in 60 women with AUFI in France, which found that 58.3% would partake in a UTx clinical trial.^80^

Given the additional risks associated with UTx, current selection criteria for a continuing UK research trial using deceased donors (Investigational Study Into Transplantation of the Uterus; INSITU) ensure recipients are aged 24–38, have a BMI <30 kg/m^2^ and normally functioning ovaries.^81^ Exclusion criteria include already having children, poor fitness and health or significant medical or psychiatric comorbidity, major or multiple previous abdominal surgery, or severe endometriosis.^81^ Moreover, potential recipients with a previous history of cancer must have been in remission for at least 5 years, owing to the risk of recurrence during this high‐risk period^82^ when immunosuppression is commenced. Ethical and legal reasons mean it is likely that many of these selection criteria will be alleviated following transition into clinical practice;^83^, ^84^ nevertheless, the selection criteria utilised to optimise success and safety will continue to restrict UTx availability among potential recipients.

## Management

In most cases, the diagnosis of AUFI is unexpected and can be highly traumatising, particularly when a woman has not yet completed her reproductive plans. Women with congenital causes, such as MRKH or other uterine anomalies, are often managed in specialist tertiary referral centres, where team members are experienced at sensitive diagnosis disclosure, arranging appropriate counselling and psychological support and offering management to optimise sexual function in those with suboptimal vaginal length.^85^, ^86^ Given the rapid progress and demand for UTx among women with AUFI, and considering the anticipated transition into clinical care, the potential impact of the vaginal restoration method on future suitability for UTx should be contemplated. While dilator therapy,^86^ or the Vecchietti procedure,^87^ would create a physiologically functioning mucosal vagina, the creation of a neovagina using skin, peritoneum or intestine would probably create a dysbiotic environment that might affect future clinical and reproductive outcomes following UTx.^88^ As such, some UTx programmes currently exclude women with intestinal neovagina from undergoing UTx.^81^

MRKH is traditionally considered a sporadic condition, owing to previously reported discordance between identical twins^89^ and the fact no females with MRKH have been born from surrogate pregnancies using oocytes from women with MRKH.^90^, ^91^ However, familial cases have more recently been reported involving both males and females.^92^, ^93^ Recent advancement in sequencing technologies has revealed the partially genetic makeup of MRKH.^94^, ^95^, ^96^ As such, genetic counselling is essential for women who wish to undergo surrogacy or UTx. In suspected familial cases, exome sequencing, or adoption, should be considered.

**Figure 2:**
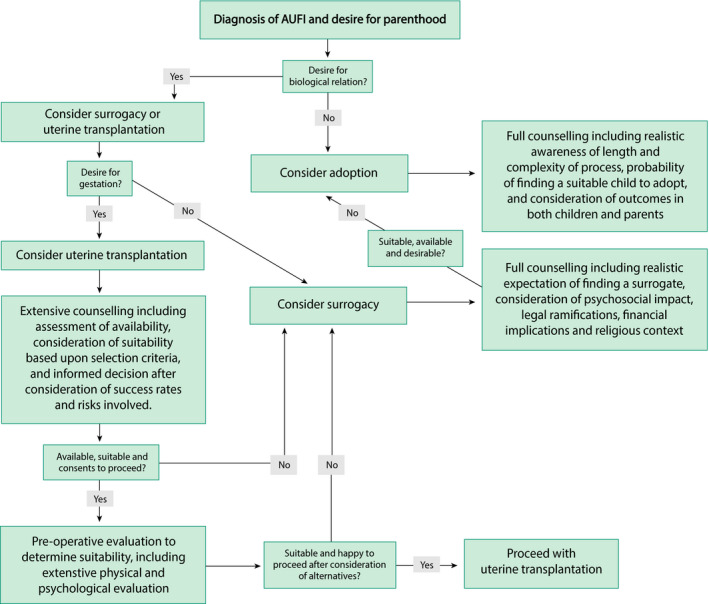
Suggested management algorithm for options to acquire motherhood in women with absolute uterine factor infertility. AUFI = absolute uterine factor infertility

Women with acquired causes of AUFI who have not yet completed their family, such as cases of emergency hysterectomy or development of Asherman’s syndrome, require similar reproductive counselling to those with congenital causes. It is essential to explore reproductive aspirations and to fully inform such women at the earliest opportunity so that realistic reproductive plans can be made in the context of their options. A suggested – albeit simplified – management algorithm is demonstrated in Figure 2. All women should receive extensive reproductive counselling about the options available to them, considering the advantages and disadvantages (as summarised in Table 1), including the associated legal and financial implications. Women who do not desire biologically related offspring ought to consider adoption. For those for whom biological relation is important, surrogacy and UTx should be primarily pursued, considering the limitations associated with surrogacy and the extensive selection criteria and risks involved with UTx. In such women, the implications of age upon ovarian reserve should be discussed, considering oocyte or embryo cryopreservation before the physiological decline in oocyte quality and quantity,^97^ to optimise future chances of success.

**Table 1:** Advantages and disadvantages of the options for parenthood in women with absolute uterine factor infertility

Option for parenthood	Advantages	Disadvantages
Adoption	Acquires social and legal parenthood Provides opportunity to enhance the life of a less fortunate child, with subsequent better psychological outcomes, especially if adopted earlier^19^, ^20^, ^21^ Generally positive outcomes; three‐quarters of adoptive parents report adoption had a positive effect on family^25^, ^26^	Lengthy process involving extensive formal evaluation^22^ Potential for increased anxiety if not able to bond with child^23^, ^24^ Challenging process: approximately 1 in 10 adoptions report breaking down and one‐quarter report finding it difficult^28^ Risk of disruption to current family unit
Surrogacy	Allows biological relation to child Following successful completion of parental order, legal parenthood is obtained Excellent perinatal and long‐term psychological outcomes in children, comparable to oocyte donation^37^, ^45^ Excellent outcomes for intended parents, with similar psychological outcomes compared with natural conception^37^ More than one child can be attained, if relationship with surrogate remains positive, with the possibility of a second sibling	Ethical/cultural/religious barriers Legal prohibitions in many countries curtail availability^34^ In the UK, the surrogate is legally recognised as the mother at birth despite origin of the gametes and contractual agreements Small transient risk of surrogate finding relinquishing care difficult^46^ Increased anxiety for intended parents: potential for surrogate not transferring parental rights after birth of child High costs: UK £25,000; USA £120,000^39^
Uterine transplant	Restores reproductive function, enabling the woman to experience gestation and childbirth Allows biological relation to child Automatically considered legal parents Widely accepted across the main cultural/religious groups More than one child can be attained with the possibility of a second pregnancy	Significant surgical risks related to 3–4 open surgeries Immunosuppression risks related to transient use while graft in situ Risk of failure: one‐quarter require emergency hysterectomy^12^ Exposure of additional risk to a second individual if using a living donor Strict selection criteria curtail availability High financial cost: Europe €100,000^78^

## Conclusion

At present, nearly all women with AUFI face a choice between involuntary childlessness and acquiring parenthood through adoption or surrogacy. The need for adoption continues to rise, with an annually increasing number of children in need of a permanent home. However, while undoubtedly beneficial for most adopted children and parents, the absence of a biological relationship, along with potential emotional, behavioural and relational issues, mean that prospective parents must think carefully about this option. Surrogacy offers a chance to have biologically related offspring, its outcomes are generally positive and multiple attempts are possible, thereby opening up the possibility for siblings in the future. However, in many jurisdictions, its legal position is problematic, which can cause uncertainty for, or make it difficult to commission, surrogates without going overseas. In addition, some cultures or families may reject surrogacy because of ethical or religious beliefs that surrogacy is unacceptable. More than 70 UTx cases have now been undertaken and, following at least 18 live births after successful procedures, UTx is now considered a feasible fertility‐restoring treatment for women with AUFI. However, it is associated with considerable surgical and immunosuppressive‐related risk and, based on cases performed so far, a >25% risk of unplanned hysterectomy. The choices faced by women with AUFI are complex and sensitive. Women’s beliefs and preferences regarding parenthood are often rooted in, and engage with, deeply held aspirations and values. Extensive reproductive counselling is therefore essential for women with AUFI, in the context of collaborative multi‐disciplinary care, to raise awareness of their options to acquire motherhood and the associated advantages and disadvantages each option presents.

### Disclosure of interests

ND sits on the Ethics Board of Surrogacy UK. JRS is the Chair of Womb Transplant UK.

### Contribution to authorship

BJ instigated and wrote the manuscript. NRZ, SV, NW and SW assisted in writing the manuscript. MYT, MAM, ND, GR, GT, LJ, JY and JRS provided guidance and helped revised the final draft. All authors approved the final version of the manuscript.
